# Supplementary material for: MD-DFT Calculations on Dissociative Absorption Configurations of FOX-7 on (001)- and (101)-Oriented Crystalline Parylene Protective Membranes
Source: Polymers (Basel). 2024 Feb 5;16(3):438. doi: 10.3390/polym16030438 (PMC10857406; doi:10.3390/polym16030438)
Supplement: Supplementary file 1 [file polymers-16-00438-s001.zip › polymers-2262090-supplementary.pdf]

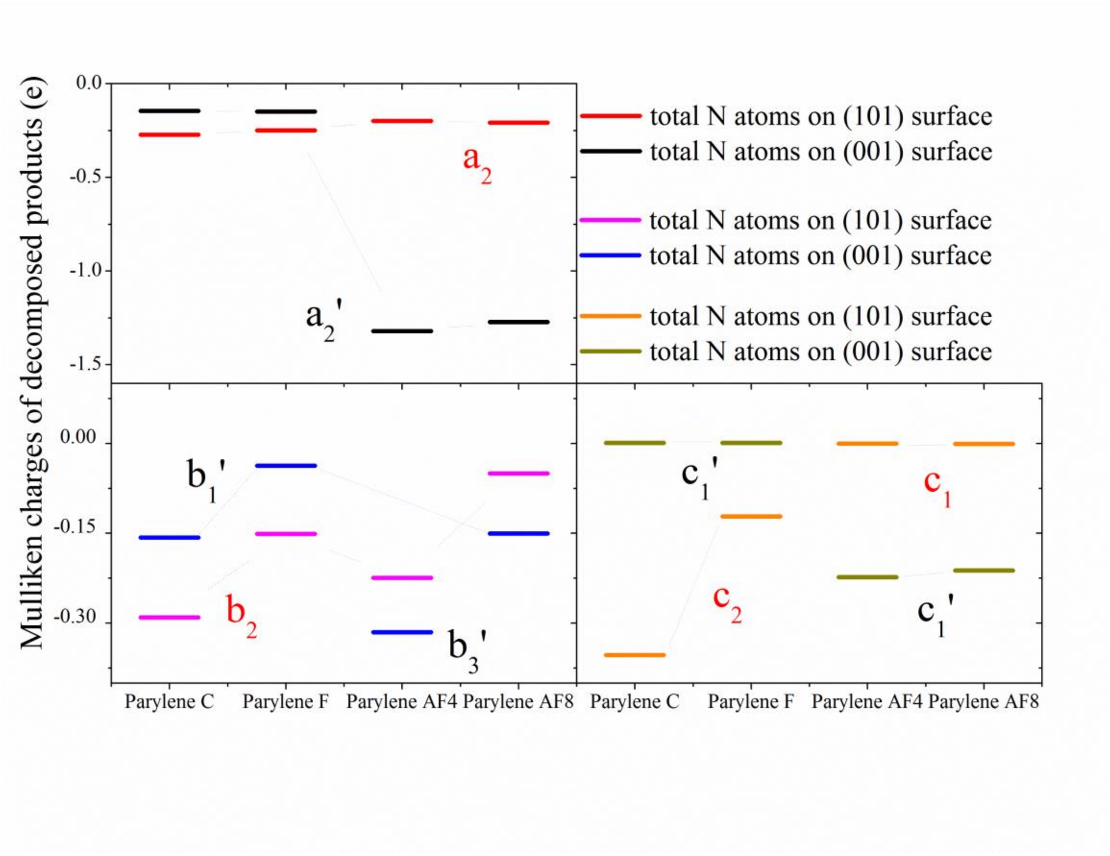

Figure S1. Mulliken charges of total N atoms of FOX-7's decomposition products on (101) and (001) orientated parylene membranes.
